# Treatment of Cryptogenic Stroke Patients with Atrial Fibrillation Detected by Insertable Cardiac Monitors Reduces Recurrent Stroke Risk to Background Levels

**DOI:** 10.19102/icrm.2021.121204

**Published:** 2021-12-15

**Authors:** E. Martin Kloosterman, Jonathan Z. Rosman, Eric J. Berkowitz, Murray Rosenbaum, Zachary A. Wettenstein

**Affiliations:** ^1^Cardiac Arrhythmia Services, Florida Atlantic University CESCOM, Boca Raton, FL, USA

**Keywords:** Atrial fibrillation, cryptogenic stroke, guided oral anticoagulation therapy, implantable cardiac monitor, stroke reduction

## Abstract

Atrial fibrillation (AF) is a known risk factor of ischemic stroke with a reported fivefold increase in incidence. However, it is not well established whether treatment with oral anticoagulation (OAC) in cryptogenic stroke patients with AF, detected by insertable cardiac monitors (ICMs), reduces the risk of recurrent stroke. We aimed to compare recurrent stroke rates between cryptogenic stroke patients who have AF detected by ICMs and thus started on OAC treatment and those without detected AF. We performed a combined retrospective and prospective analysis of consecutive patients who received an ICM indicated for cryptogenic stroke and were followed up with between July 2015 and November 2019. Patients with a prior documented history of AF were excluded. All patients were required to have a home remote monitoring system. We calculated the rates of AF detection and OAC initiation, then compared recurrent annualized stroke rates (ASRs) between patients with and without AF detected. A total of 298 patients with ICMs were included in the study [mean ± standard deviation age: 77 ± 11.7 years; female/male: 147/151; virtual CHA_2_DS_2_-VASc score: 4.96 ± 1.28 points]. AF was discovered in 91 patients (~30%) over a mean 19.3 months follow-up. Of those, 65 (71.4%) were started on OAC, 12 (13.2%) were already on OAC, and 10 (11%) remained non-anticoagulated. In four (4.4%) patients, OAC was started after recurrent stroke when AF was diagnosed. A total of 24 of 298 patients developed recurrent strokes (ASR: 5.0%). Among the 24 patients with recurrent strokes, four had new AF and were on OAC (ASR: 3.23%), six had new AF and were not anticoagulated (ASR: 26.62%), and 14 had no AF detected and no OAC (ASR: 4.20%). Our study found new AF detected by ICMs in almost one-third (30%) of cryptogenic stroke patients (consistent with previous studies), and the majority of them (89%) received OACs. There was no significant difference in the recurrent stroke rate among patients without AF detected and those with AF detected and on OAC. Rigorous arrhythmia monitoring using ICMs can increase new AF detection rates in cryptogenic stroke patients, thereby allowing early initiation of OACs, ultimately reducing the risk of recurrent stroke to background levels.

## Introduction

Atrial fibrillation (AF) is a known risk factor of ischemic stroke with a reported fivefold increase in incidence.^1^ However, it is not well established whether treatment with oral anticoagulation (OAC) in cryptogenic stroke patients with AF, detected by insertable cardiac monitors (ICMs), reduces the risk of recurrent stroke.

Stroke is the second leading cause of disability and the second leading cause of death worldwide.^2^ It is estimated that 795,000 people experience a stroke each year, with approximately 185,000 of those being recurrent strokes.^3^ In the United States, approximately 87% of strokes each year are ischemic in origin, and, of those, it is estimated that more than 25% of them are cryptogenic in nature,^3–5^ simply meaning that, despite diagnostic workup, no definitive origin or cause could be found for the incident.

The Asymptomatic AF and Stroke Evaluation in Pacemaker Patients and the AF Reduction Atrial Pacing Trial (ASSERT) and Cryptogenic Stroke and Underlying AF (CRYSTAL AF) trial^5,6^ found that a significant proportion of patients with cryptogenic stroke have silent undiagnosed paroxysmal atrial fibrillation (PAF), which is not temporally related to a neurological event.^7^ As such, it became clear that long-term cardiac monitoring increases the yield of newly diagnosed PAF in patients with cryptogenic stroke. These patients were thereafter started on OAC. Although a potential benefit was suggested by a meta-analysis,^8^ a specific assessment has yet to be done on whether new AF discovery and OAC treatment actually affect the outcome, providing protection from a recurrent stroke and further overall morality. Yet, it has been shown that patients found to have AF do have a 10% to 20% reported risk of reoccurrence over the next year.^9^

However, it should be noted that it has been established that preemptive OAC (without an AF diagnosis) after cryptogenic stoke is counterproductive.^10,11^

We aimed to compare recurrent stroke rates between cryptogenic stroke patients who have AF detected by ICMs and thus started on OAC treatment and those without detected AF.

## Methods

### Study design

We performed a single-center, combined retrospective/prospective cohort analysis of consecutive patients who received an ICM by our service indicated for cryptogenic stroke between July 2015 and November 2019. Patients with cryptogenic stroke who received an ICM (Reveal LINQ™; Medtronic Inc., Minneapolis, MN, USA) implant and were followed up by our service using home remote monitoring via the CareLink™ network (Medtronic) were included, while patients with a prior documented AF history were excluded.

All patients’ data were deidentified after demographic information was recorded. An analysis of patients’ data was then performed. The patients’ stroke history was confirmed, cardiac history was assessed to evaluate if the patients had a prior history of AF, past medical history was reviewed to obtain the CHA_2_DS_2_-VASc score, and the medication list was checked for baseline OAC. The LINQ™ data were then reviewed for evidence of AF. AF diagnosis for this study required at least a two-minute arrhythmia duration for automatic alert by the LINQ™ device, in addition to confirmation and formal diagnosis adjudication by the clinician. The majority of patients had in-office follow-up appointments, and all patients were contacted via phone, at a minimum, when a diagnosis was made. The patients were divided into two different cohorts based on whether AF was discovered or not. The patients were then evaluated for recurrent strokes via records of hospital emergency department visits, hospital admissions, and neurology office visits. The criteria for a recurrent stroke/transient ischemic attack were any clinical neurological evaluation due to altered mental status, weakness, dysphagia, or visual changes not found to be caused by another underlying disorder or etiology.

### Patients

The subjects’ pool meeting inclusion criteria consisted of 298 patients who received a Medtronic LINQ™ ICM device and were enrolled under monitoring services between July 2015 and March 2019. The patients’ mean age was 77 years. The male-to-female ratio was almost equal at 151 to 147 **(Table 1)**.

Patients participated in three to six months of in-office follow-ups. All patients also were subjected to 24-hour home monitoring with bedside remote monitoring for symptom triggers, nightly and weekly alerts, and monthly uploads, as well as in-office download transmission at in-person follow-up.

### Statistical analysis

Odds ratios and relative risk values were used as measures of association between groups.

## Results

A total of 298 patients were monitored with a mean follow-up post-implant of 19.3 months [mean ± standard deviation age: 77 ± 11.7 years; female/male: 147/151]. The total group CHA_2_DS_2_-VASc score was calculated as 4.96 ± 1.28 points, even though not all patients had PAF; the score was similar in all individual groups. Patient demographic details can be seen in **Table 1**.

AF was newly diagnosed in 91 (~30%) patients. Of these 91 patients, 77 were anticoagulated; 12 (13.2%) were already on OAC and 65 (71.4%) were started on OAC after AF detection. Of note, four patients were only found to have AF and were anticoagulated after a recurrent stroke event occurred. Of the 91 patients with newly diagnosed PAF, 10 (11%) were not anticoagulated. The reasons for no OAC were: three had left atrial appendage (LAA) clips; one had undergone coronary artery bypass grafting, Cox maze surgery, and LAA exclusion; four were given OACs but were noncompliant; one refused; and one had AF detected after recurrent stroke with hemorrhagic conversion preventing the use of OAC.

**Figure 1:**
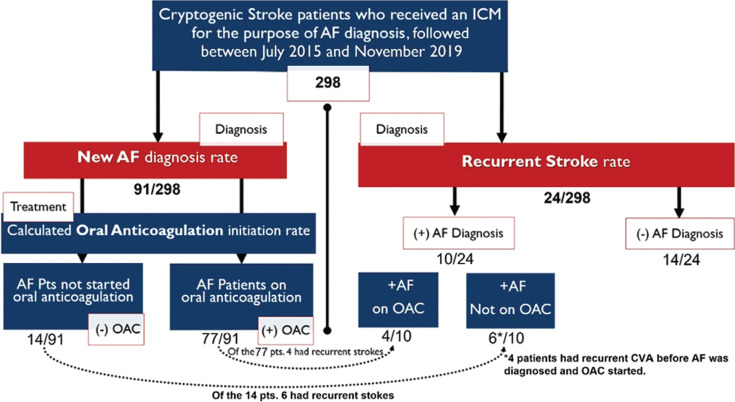
Flowchart showing the distribution of the 298 patients enrolled with cryptogenic stroke and ICM implant for the purpose of diagnosing silent AF. Two main cohorts are depicted: one of new AF diagnosis and another of recurrent stroke. The relationship between the two groups is illustrated. AF: atrial fibrillation; ICM: insertable cardiac monitor; OAC: oral anticoagulation.

The average time from ICM implant to AF diagnosis was 201 days, with an average duration of the first event being 24 minutes and the shortest being two minutes **(Figure 1)**.

A total of 24 patients developed recurrent strokes for an annualized stroke rate (ASR) of 5.0%. Of those, four strokes occurred among the 77 patients with newly detected AF and subsequent anticoagulation, for an ASR of 3.23%. Four patients were found to have reoccurrences before AF was detected on the ICM, so they were undiagnosed and untreated despite an underlying AF. Therefore, they were not anticoagulated at the time of their recurrent stroke. Of the 10 patients found to have AF but not anticoagulated, two had recurrent strokes. As both sets of patients technically had AF with no OAC, they were grouped together. This resulted in six recurrent strokes in 14 patients, for an ASR of 26.62%. The remaining 14 recurrent strokes occurred among the 207 patients without AF detected, for an ASR of 4.20% **(Figure 1)**.

In patients with an AF diagnosis, not including the four patients with recurrent cerebrovascular accident (CVA) prior to detection/diagnosis, the average time from AF to recurrent stroke was 22.2 months.

Three patients with newly diagnosed AF on no OAC did have an LAA clip device. One of them had a recurrent stroke despite this but refused the recommended OAC.

The shortest time from ICM implant to recurrent stroke was 10 days and the longest was 1,073 days (~35 months), with a mean time from ICM implant to recurrent stroke of 193 days (6.4 months).

**Figure 2:**
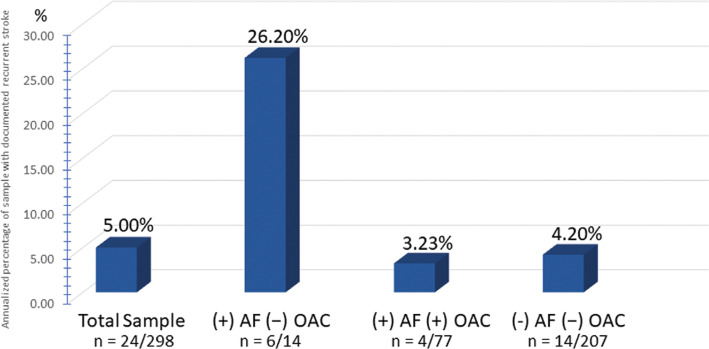
Comparison of the annualized recurrent stroke rates in patients with prior cryptogenic stroke between the total sample versus patients diagnosed with AF on no OAC, patients with AF on treatment with OAC, and those with no AF and therefore no OAC treatment. AF here refers to newly diagnosed AF during the study observation period. AF: atrial fibrillation; OAC: oral anticoagulation.

The average length of follow-up for the study was 19.3 months, which was used in the calculations to find the ASR **(Figure 2)**.

No hemorrhagic strokes were reported in the AF with OACs group, except three minor bleeding instances (two gastrointestinal, one urinary), and all patients were eventually restarted on OACs.

## Discussion

This is a combined retrospective/prospective, single-center, nonrandomized study with limitations, such as the relatively small size of the secondary comparison group of AF without OAC. A larger sample and longer follow-up time would have also been desirable, but, as a point of reference, the seminal CRYSTAL AF trial^5^ involved 441 patients with a follow-up of 12 months. The results of our study regarding the incidence of newly diagnosed PAF in patients post-cryptogenic stroke are in line with those findings, as is the rate of OAC treatment thereafter.^5,6^

The annualized risk of recurrent strokes in patients with AF not on OAC was at least fivefold greater, as compared to the incidence of stroke in non-AF and AF patients treated with OAC, which is also consistent with previous publications.^1,12^ The study population had a mean CHA_2_DS_2_-VASc score of 4.9 points and a mode score of five points, at an ASR of 5.0%, consistent with the expected stroke risk.^13–15^ Additionally, all cohorts had an individual average CHA_2_DS_2_-VASc score between 4 and 6. We did not stratify or collect data based on ethnicity. However, race/ethnicity has not been found to be a reliable individual predictor of stroke and has been shown to have no effect on CHA_2_DS_2_-VASc risk stratificiation.^16^

The burden of AF detected on the ICM was relatively low in patients with AF who had a recurrent stroke. Excluding the patient who was in persistent AF as an outlier, the average device burden of the group was 6% **(Table 2)**. Current research is underway to evaluate the effect of AF burden on stroke risk. The Kaiser Permanente Real-world Heart Monitoring Strategy Evaluation, Treatment Patterns, and Heart Metrics in AF (KP-RHYTHM) study associated a burden of greater than 11% with an increased risk of thromboembolic events.^17–19^ Our overall average of patients with AF and recurrence is above this threshold at 15%; however, as already mentioned, after correcting for the outlier, the burden is only 6%. Also, as previously mentioned and supported by our data, initiation of proper OAC should effectively neutralize the risk of AF regardless of burden, thus making it hard to evaluate or comment on this effect. However, it is interesting to note that of the patients not on OAC (due to not-yet-diagnosed AF), they appeared to have a majority of burden less than 1% **(Table 2)**.

When compared, the 95% confidence interval (CI) of the “non-AF group” and “AF-OAC group” odds ratio was 0.25 to 2.3, supporting the notion that our “AF-OAC group” is back to baseline recurrent stroke levels, as there was no significant difference between the two groups. We did not see significant risk reduction from baseline (no AF) in the AF-OAC group, evidenced by the 95% CI of the relative risk from 0.33 to 2.3. However, our goal was not to show improvement of recurrent stroke from the baseline of the non-AF group. We thought that once AF was an effectively “neutralized risk” through OAC, cohorts were expected to have similar functions of risk and therefore equivocal rates of recurrent stroke, demonstrated through near-equal average CHA_2_DS_2_-VASc scores across the board.

When the stroke rates between the AF groups that received OAC versus those who did not were compared, there was a significant decrease in the likelihood of recurrence in the OAC group (odds ratio of 0.07 with 95% CI from 0.017 to 0.309). This finding correlated exposure to OAC with a reduction in recurrent stroke in patients with AF. As such, it was viewed as a secondary outcome measure that further supported the primary outcome of return to baseline.

On this note, it is important to reiterate the concept of time independence between AF event and stroke as shown by the ASSERT investigators.^5^ In our cohort, four patients had recurrent strokes manifested before AF was diagnosed, only allowing for the initiation of anticoagulation after the recurrent CVA occurred. Although one could argue that these are completely independent situations, there is the notion of underlying atrial myopathy as a substrate for clot formation and AF.^13,20^ After anticoagulation, all four patients remained stroke-free for the duration of the study: 345, 378, 546, and 950 days, further emphasizing the importance of early recognition of AF in these patients.

Lastly, we recognized that the analysis of those patients (n = 4) with LAA exclusion could have been performed in different ways as the population was small and, for the purposes of this study, we chose OAC as the standard to separate cohort for stroke prevention.

To the best of our knowledge, this is the first study able to specifically prove that the use of ICM in cryptogenic stroke is not only helpful to diagnose silent AF but also allows guided OAC therapy to effectively reduce the risk of recurrent stroke.

## Conclusions

Our study found that new AF was detected by the ICM in almost one-third (30%) of cryptogenic stroke patients (consistent with previous studies), and the vast majority (89%) received OAC thereafter. There was no significant difference in the recurrent stroke rate among patients without AF detected and those with AF detected and on OAC, supporting that our patients returned to baseline risk. Rigorous arrhythmia monitoring using ICMs can increase new AF detection rates in cryptogenic stroke patients, thereby allowing early initiation of OAC, ultimately reducing the risk of recurrent stroke to background levels.

## Figures and Tables

**Table 1:** Demographics of Patients in the Two Study Cohorts

	Recurrent Stroke		Atrial Fibrillation	
	Yes	No	Yes	No
Patients per group	24	274	91	207
Sex				
Male	11	140	46	105
Female	13	134	45	102
Age, years				
< 40	0	1	0	1
40–50	0	6	1	5
51–60	0	17	3	14
61–70	2	28	7	23
71–80	10	80	30	59
> 80	12	142	50	105
CHA_2_DS_2_-VASc score, points				
0–1	0	0	0	0
2	0	12	4	8
3	1	27	6	22
4	3	54	13	44
5	7	80	27	60
6	10	78	33	55
7	3	22	7	18
8	0	1	1	0
Average score	5.5	4.9	5	5.5
Primary risk factors, n (%)				
Hypertension	16 (66.67%)	172 (62.77%)	54 (59.34%)	124 (59.90%)
Diabetes mellitus	8 (33.33%)	55 (20.07%)	16 (17.58%)	47 (22.71%)
Coronary artery disease	7 (29.17%)	40 (14.60%)	18 (19.78%)	28 (13.53%)

Note that all patients in the study had a baseline stroke (not listed as a risk factor), adding two points to the CHA_2_DS_2_-VASc score.

**Table 2:** AF Burden and Associated Data Compilation of the 10 Patients Newly Diagnosed with AF Who Experienced Recurrent Stroke

Patient ID	Days from ICM to AF Diagnosis	OAC?	Days from ICM to Recurrent Stroke	Days from AF to Recurrent Stroke	First AF Duration, min	AF Burden
175	76	Yes	1073	997	120	0.1%
99	107	Yes	661	554	350	2.2%
212	126	No	130	4	Persistent	100%
93	139	No	26	After	420	27.3%
28	330	No	35	After	2	0.1%
2	407	No*	588	181	128	1.0%
67	451	Yes	801	350	4	17.5%
72	640	No	310	After	18	0.3%
50	676	Yes	852	176	22	5.6%
9	815	No	240	After	2	0.2%
Average burden						15%
Average burden excluding persistent AF						6%

AF: atrial fibrillation; ICM: insertable cardiac monitor; OAC: oral anticoagulation.

*This patient had an LAA clip.

## References

[1] Wolf PA, Abbott RD, Kannel WB (1991). Atrial fibrillation as an independent risk factor for stroke: the Framingham study. Stroke.

[2] GBD 2016 Neurology Collaborators (2019). Global, regional, and national burden of neurological disorders, 1990–2016: a systematic analysis for the Global Burden of Disease Study 2016. Lancet Neurol.

[3] Virani SS, Alonso A, Benjamin EJ (2020). Heart Disease and Stroke Statistics-2020 Update: a report from the American Heart Association. Circulation.

[4] Li L, Yiin GS, Geraghty OC (2015). Incidence, outcome, risk factors, and long-term prognosis of cryptogenic transient ischemic attack and ischemic stroke: a population-based study. Lancet Neurol.

[5] Brambatti M, Connolly SJ, Gold MR (2014). Temporal relationship between subclinical atrial fibrillation and embolic events. Circulation.

[6] Sanna T, Diener HC, Passman RS (2014). Cryptogenic stroke and underlying atrial fibrillation. N Engl J Med.

[7] Daoud EG, Glotzer TV, Wyse DG (2011). Temporal relationship of atrial tachyarrhythmias, cerebrovascular events, and systemic emboli based on stored device data: a subgroup analysis of TRENDS. Heart Rhythm.

[8] Tsivgoulis G, Katsanos AH, Grory BM (2019). Prolonged cardiac rhythm monitoring and secondary stroke prevention in patients with cryptogenic cerebral ischemia. Stroke.

[9] Saxena R, Lewis S, Berge E, Sandercock PA, Koudstaal PJ (2001). Risk of early death and recurrent stroke and effect of heparin in 3169 patients with acute ischemic stroke and atrial fibrillation in the International Stroke Trial. Stroke.

[10] Diener H-C, Sacco RL, Easton JD (2019). Dabigatran for prevention of stroke after embolic stroke of undetermined source. N Engl J Med.

[11] Hart RG, Sharma M, Mundl H (2018). Rivaroxaban for stroke prevention after embolic stroke of undetermined source. N Engl J Med.

[12] Kannel WB, Benjamin EJ (2008). Status of the epidemiology of atrial fibrillation. Med Clin North Am.

[13] Gažová A, Leddy JJ, Rexová M, Hlivák P, Hatala R, Kyselovič J (2019). Predictive value of CHA2DS2-VASc scores regarding the risk of stroke and all-cause mortality in patients with atrial fibrillation (CONSORT compliant). Medicine (Baltimore).

[14] Lip GYH, Nieuwlaat R, Pisters R, Lane DA, Crijns HJGM (2010). Refining clinical risk stratification for predicting stroke and thromboembolism in atrial fibrillation using a novel risk factor-based approach: the euro heart survey on atrial fibrillation. Chest.

[15] Senoo K, Lane D, Lip GY (2014). Stroke and bleeding risk in atrial fibrillation. Korean Circ J.

[16] Chen LY, Norby FL, Chamberlain AM (2019). CHA2DS2-VASc score and stroke prediction in atrial fibrillation in whites, blacks, and Hispanics. Stroke.

[17] Go AS, Reynolds K, Yang J (2018). Association of burden of atrial fibrillation with risk of ischemic stroke in adults with paroxysmal atrial fibrillation: the KP-RHYTHM study. JAMA Cardiol.

[18] Chen LY, Chung MK, Allen LA (2018). Atrial fibrillation burden: moving beyond atrial fibrillation as a binary entity: a scientific statement from the American Heart Association. Circulation.

[19] Passman R, Bernstein RA (2016). New appraisal of atrial fibrillation burden and stroke prevention. Stroke.

[20] Packer M (2020). Characterization, pathogenesis, and clinical implications of inflammation-related atrial myopathy as an important cause of atrial fibrillation. J Am Heart Assoc.

